# From Parent to Child to Parent: Associations Between Parent and Offspring Psychopathology

**DOI:** 10.1111/cdev.13402

**Published:** 2020-08-26

**Authors:** Yllza Xerxa, Leslie A. Rescorla, Jan van der Ende, Manon H.J. Hillegers, Frank C. Verhulst, Henning Tiemeier

**Affiliations:** ^1^ Erasmus University Medical Center; ^2^ Bryn Mawr College; ^3^ University of Copenhagen; ^4^ Harvard TH Chan School of Public Health

## Abstract

Parental psychopathology can affect child functioning, and vice versa. We examined bidirectional associations between parent and offspring psychopathology in 5,536 children and their parents. We asked three questions: (a) are parent‐to‐child associations stronger than child‐to‐parent associations? (b) are mother‐to‐child associations stronger than father‐to‐child associations? and (c) do within‐ and between‐person effects contribute to bidirectional associations between parent and offspring psychopathology? Our findings suggest that only within‐rater bidirectional associations of parent and offspring psychopathology can be consistently detected, with no difference between mothers and fathers. Child psychopathology was hardly associated with parental psychopathology. No evidence for cross‐rater child‐to‐parent associations was found suggesting that the within‐rater child‐to‐parent associations reflect shared method variance. Moreover, within‐person change accounted for a part of the variance observed.

Parental psychopathology has been found to increase risk for a wide range of negative mental health outcomes, including child internalizing and externalizing problems (Connell & Goodman, 2002). The recognition of the importance of bidirectional associations in the transactions between parents and children is often attributed to Sameroff and Chandler (1975). Further advocacy of the significance of the bidirectional associations in child development was also provided by Sameroff (1975), who argued for a transactional model of development whereby the relationships between children and their caregivers “change, maintain, and then change again the characteristics of participants” (Sameroff, 1975, p. 3). Not only do behaviors of parents impact behaviors of their children, but children’s behaviors also impact behaviors of their parents (Pettit & Arsiwalla, 2008). In other words, children’s adjustment problems reflect the continuous interplay between individual characteristics that children bring to their social interactions and the quality of social support and resources (Bell, 1968; Cicchetti, Toth, Luthar, Burack, & Weisz, 1997; Sameroff, 2009). Despite the fact that the bidirectional, theory of child development is now at least 50 years old, much research still regards children as the passive recipients of their parents’ socialization (O'Connor, 2002; Perlman & Ross, 1997). To help address this limitation of much prior research, our study adopted a bidirectional approach. Using a large and diverse population sample, we analyzed data from a 10‐year longitudinal study to test bidirectional associations between self‐reported parental psychopathology and parent‐rated child psychopathology at multiple time points. The study of bidirectional transactional associations between parents and children is consistent with a developmental psychopathology perspective of bidirectional influences on development (Cowan & Cowan, 2006; Sameroff & Fiese, 2000).

## Previous Studies of Bidirectional Associations in Parent and Child Psychopathology

Bidirectional associations have been studied with respect to child psychopathology and parenting practices such as dysfunctional parenting (Childs, Fite, Moore, Lochman, & Pardini, 2014; Combs‐Ronto, Olson, Lunkenheimer, & Sameroff, 2009; Pardini, Fite, & Burke, 2008; Shaffer, Lindhiem, Kolko, & Trentacosta, 2013). Taken together, these studies found some evidence for a bidirectional association between parenting and child problems. Typically, maternal to child associations were stronger than paternal to child associations, although most studies relied on maternal reports only. Therefore, many studies that investigated the association between parental psychopathology and child psychopathology have primarily focused on the unidirectional relation from parent to child. These studies showed that parental psychopathology adversely affects child problem behavior, including depression, anxiety, and aggression (Breaux, Harvey, & Lugo‐Candelas, 2014; Joelsson et al., 2017; Middeldorp et al., 2016; Nath, Russell, Kuyken, Psychogiou, & Ford, 2016). The exposure to parent psychopathology may place children at risk for internalizing and externalizing problems through a number of processes. These processes include shared genetics, disruptions in parenting, exposure to parents’ maladaptive cognitions, affect, and behavior (Dodge, 1990; Goodman & Gotlib, 1999), as well as exposure to stressful life‐events and lack of parental social support (McCarty & McMahon, 2003). However, children also play an active role in influencing their parents’ behavior and well‐being (Pardini, 2008). Although less is known about the impact of child problem behavior on parental psychopathology, evidence suggests that children’s problem behavior, especially disruptive behavior, is likely to be associated with parental psychopathology (Forbes et al., 2008; Goodman & Gotlib, 1999). Much less is known about the bidirectional relationship between child problem behavior and parental psychopathology, such as depression and anxiety (Gross, Shaw, Moilanen, Dishion, & Wilson, 2008).

We were interested in the reciprocal associations between parental psychopathology and problem behavior in children prior to adolescence. In the first decade of life, influences on child behavior usually are confined to the family context and to the school, with less influence from the wider environment, especially peers, than is the case with adolescents.

To our knowledge, there are only a few studies on bidirectional associations between parental psychopathology and problem behavior in preschool or school‐aged children. These studies give a mixed picture. For example, Gross, Shaw, and Moilanen (2009) and Gross, Shaw, Burwell, and Nagin (2008) provided some support for bidirectional associations between parental depression and adolescent disruptive behaviors. However, Gross, Shaw, Moilanen, et al. (2008) did not provide support for reciprocal associations between child and parental psychopathology in preschoolers. They found that maternal and paternal depressive symptoms at child age 2 predicted child internalizing problems at age 4, but no reverse association in early childhood.

Nicholson, Deboeck, Farris, Boker, and Borkowski (2011) reported bidirectional associations of maternal depressive symptoms and child internalizing and externalizing problems based on mother reports only. The authors indicated that mother‐to‐child influences were greater than child‐to‐mother influences. Choe, Olson, and Sameroff (2014) provided no support for bidirectional associations between maternal depression and child externalizing behavior using mother‐reported depressive symptoms and teacher‐reported child externalizing problems. However, when allowing for the moderating effects of gender and the level of children’s effortful control (EC), Choe et al. (2014) found that child externalizing problems at age 3 were associated with fewer depressive symptoms in mothers of children with high EC at child age 10 years. In another study, Antúnez, la Osa, Granero, and Ezpeleta (2018) found some support for bidirectional longitudinal associations between paternal but not maternal anxiety‐depression and oppositional defiant disorder problems in boys at age 3 but not in girls. Finally, in a study of adopted children and their adoptive parents, Brooker et al. (2015) showed reciprocal longitudinal associations between parental anxiety and infant negative affect, assessed by mothers’ and fathers’ reports as well as by observations of interactions with the child. Genetic liabilities from birth parents did not explain the observed associations.

The few existing studies on bidirectional associations between parental psychopathology and child problem behavior at preschool and school age thus provide some support for the presence of reciprocal associations between parental psychopathology and child problem behavior over time, but these studies have a number of limitations. First, most studies used small or selected samples across rather limited time periods, usually spanning infancy or early childhood. Few studies involved unselected samples of children from the general population over wide enough time intervals to cover major developmental periods and test the stability of bidirectional effects of parental and child psychopathology over time. Second, results are inconclusive with regard to the level of symmetry in the bidirectional associations between parent and child (Choe et al., 2014; Nicholson et al., 2011), with only some indicating that parent to child influences were greater than child to parent influences (Gross, Shaw, Moilanen, et al., 2008). Third, a particularly notable limitation of literature is that the extant research has primarily focused on group‐level differences (i.e., between‐person associations), thus overlooking stability and change at the individual level (i.e., within‐person associations). Understanding the variability at the individual level is likely the most relevant for developmental theory and intervention science (Curran, Howard, Bainter, Lane, & McGinley, 2014). Fourth, studies are inconsistent regarding whether these bidirectional associations are similar for maternal and paternal report of child problem behavior.

A frequently encountered problem in the study of child psychopathology is that of shared‐rater variance. When the same reporter provides ratings on the predictor and the outcome, part of the explained variance may be due to the informant who is reporting rather than to the constructs the measures are assumed to represent. For example, if mothers report on their own problems as well as on their child’s problems, there is the likelihood of halo effects in ratings, reflecting shared informant variance and therefore resulting in inflated parent‐to‐child associations. Ringoot et al. (2015) showed that more than 30% of an effect can in some instances be attributed to this shared rater variance. Results provided support that shared rater variance affected the associations when parents reported on both their own depression and on child problem behavior, suggesting inflated associations between parental depression and child problem behavior. To avoid shared‐rater variance, information on predictor and outcome variables must be obtained from multiple sources or informants (e.g., mothers’, fathers’, teachers’ reports, children’s self‐reports).

The present study in the general population extended the literature on bidirectional associations between parent and offspring psychopathology by examining the reciprocal associations of repeatedly measured parent and offspring psychopathology up to the child’s age of 10 years. Both parents provided reports of parental psychopathology in three periods namely, the prenatal period, when the child was approximately 3 years old and approximately 10 years old. The parents each reported child internalizing and externalizing problems at ages 3 and 10. With separate measures of maternal and paternal psychopathology as well as with separate ratings of child problem behavior by mothers and fathers, we were able to examine the differences between associations of child problem behavior and parental psychopathology as assessed by the same rater versus different raters. The current study had three main aims. First, we aimed to examine bidirectional associations between parent and child psychopathology over time, and whether parent‐to‐child associations are stronger than child‐to‐parent associations. Second, we aimed to examine whether mother‐to‐child associations are stronger than father‐to‐child associations. The third aim was to disaggregate within‐ and between‐person effects in the bidirectional associations between parent and offspring psychopathology over time.

## Method

### Participants

Our study was embedded in the Generation R Study, a multi‐ethnic population‐based cohort from fetal life onwards. The Generation R Study has been described in detail previously (Kooijman et al., 2016). Briefly, all pregnant women living in Rotterdam, the Netherlands, with an expected delivery date between April 2002 and January 2006 were invited to participate. The study was approved by the Medical Ethics Committee of the Erasmus Medical Center, Rotterdam. Written informed consent was obtained from all adult participants. Of the 8,879 women enrolled during pregnancy, we excluded 1,266 mothers with no partner and 890 with missing parental psychopathology data, leaving 6,723 mothers and 5,025 fathers. For the current study, families were included if the child had behavior problem data collected at a minimum of one data point (i.e., ratings by mother at age 1.5, 3, or 10 years or by father at age 3 or 10). This resulted in 5,536 children and their parents. Self‐reported psychopathology data were missing for 21% of mothers and 33% of fathers at child age 3 and 30% of mothers and 43% of fathers at child age 10. Maternal reports of child problems were missing for 20% of the children at age 3 and 30% at age 10, whereas paternal reports of child problems were missing for 33% of children at age 3 and 45% at age 10.

### Measures

#### Parental Psychopathology

Mothers and fathers completed the Brief Symptom Inventory (BSI) to report on their psychiatric symptoms at 20 weeks pregnancy (prenatal time period, 18‐ to 25‐week gestational age) and when their child was approximately 3 and 10 years old. The BSI is a validated self‐report questionnaire with 53 items to be answered on a 5‐point scale, ranging from 0 (*not at all*) to 4 (*extremely*), (de Beurs, 2004; Derogatis & Melisaratos, 1983). At 20 weeks of pregnancy, the complete 53 item questionnaire was employed, while at 3 and 10 years a short form was used including four of nine subscales. For each measurement, we computed the Global Severity Index (de Beurs, 2004), which is the mean score of all items. The BSI is widely used instrument to measure self‐reported psychological symptoms in samples of psychiatric patients and community non‐patients. This instrument encompass three global indices and nine symptom dimensions covering clinically relevant psychiatric and psychosomatic symptoms (Derogatis & Melisaratos, 1983). High validity and reliability have been reported for the Dutch translation (De Beurs & Zitman, 2005). In the current study, internal consistencies (Cronbach’s α) ranged from .66 to .73.

#### Child Problem Behavior

The Child Behavior Checklist for toddlers (CBCL/1½–5) and for older children (CBCL/6–18; Achenbach & Rescorla, 2000, 2001) was used to obtain standardized parent reports of children’s problem behaviors. The CBCL/1½–5 contains 99 problems items, which are scored on three broadband scales (Internalizing, Externalizing, and Total Problems). The Internalizing scale comprises the Emotionally Reactive, Anxious/Depressed, Withdrawn/Depressed, and the Somatic Complaints scales, whereas the Externalizing scale comprises the Attention Problems and the Aggressive Behavior scales. Each item is scored on a 3‐point rating scale 0 (*not true*), 1 (*somewhat or sometimes true*), and 2 (*very true or often true*), based on the preceding 2 months. The CBCL/6–18 has 118 problem items, also yielding syndrome scales and the two broadband scales Internalizing and Externalizing with ratings based on the preceding 6 months. The Internalizing scale comprises the Anxious/Depressed, Withdrawn/Depressed, and the Somatic Complaints scales, whereas the Externalizing scale comprises the Rule‐Breaking Behavior and the Aggressive Behavior scales. Good reliability and validity have been reported for the CBCL/1½–5 and CBCL/6–18 (Achenbach & Rescorla, 2000). The scales were found to be generalizable across 23 societies, including The Netherlands (Ivanova et al., 2010).

We used the continuous Internalizing and Externalizing Problems scores separately rather than Total Problems score (the sum of ratings on all problem items) as our outcome measures. These broadband scales tap a wide variety of children’s emotional (Internalizing) and behavioral (Externalizing) problems. Multiple studies have documented the validity of the CBCL’s Internalizing and Externalizing scales as broadband measures of child psychopathology, starting with Achenbach (1966). Since that time, many other instruments assessing child psychopathology have adopted these broadband groups of problems (Achenbach, Ivanova, Rescorla, Turner, & Althoff, 2016). Cronbach’s alpha for the Externalizing scale ranged from .76 to .78, and for the Internalizing scale from .65 to .70.

#### Covariates

Maternal and paternal age were assessed at intake. Parental ethnicity was categorized into Dutch, non‐Western, and other Western national origin (Netherlands Statistics, 2006). Parental education was classified in three levels: “low” (maximum of 3 years general secondary school), “medium” (> 3 years general secondary school; intermediate vocational training), and “high” (Bachelor’s degree or higher academic education). Information about smoking (three categories: no smoking during pregnancy, smoked until pregnancy recognized, and continued smoking during pregnancy), alcohol intake during pregnancy (four categories: no alcohol consumption during pregnancy; alcohol consumption until pregnancy recognized; continued occasionally during pregnancy (< 1 glass/week); and continued frequently during pregnancy [1+ glass/week]) was prenatally assessed by questionnaires. Date of birth and gender of the infant were obtained from community midwife and hospital registries at birth. We controlled for potential effect of confounders, including socioeconomic factors and maternal or paternal psychopathology at baseline as they are related to parental psychopathology and/or child problem behavior (Goelman, Zdaniuk, Boyce, Armstrong, & Essex, 2014; Harvey & Metcalfe, 2012; Ramchandani & Psychogiou, 2009).

The mean age of the children was 10 years. Half (49.5%) of the children were boys. Mothers were on average 31 years at the birth of the child, fathers 33 years. In total, 28% of mothers and 25% of fathers had a non‐Western national origin. Whereas 19% of mothers and 20% of fathers had low educational level. Of mothers included in the analyses, 10.4% had actively smoked during pregnancy, whereas 7.6% of mothers continued to use alcohol during pregnancy.

### Statistical Analyses

First, we computed descriptive statistics and the correlations between parental psychopathology and CBCL Internalizing and Externalizing scores at different time points. Then, we used structural equation modeling (SEM) to test the bidirectional associations between measures of parent psychopathology (measured prenatally and at child ages 3 and 10) and child Externalizing or Internalizing problems (measured at ages 3 and 10). Prenatal maternal‐ and paternal‐reported psychopathology were included in the model because these reports are not affected by the child’s problems and could therefore be used to test associations without a possible bidirectional association. The models were adjusted for baseline potential confounders, including parental age, ethnicity, education, child sex and age, smoking, alcohol consumption, and prenatal parental psychopathology reported by mother and father.

**Figure 1:**
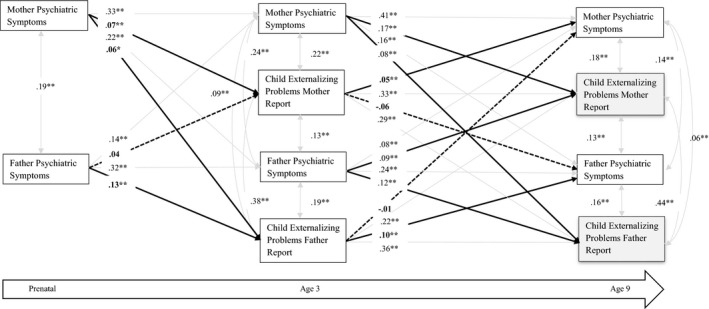
Structural equation modeling of parental psychopathology and child externalizing problems. Numeric values are standardized path regression coefficients. The models are adjusted for parental age, ethnicity, education, child sex and age, smoking, alcohol consumption and prenatal parental psychopathology reported by mother and father (root mean square error of approximation = .01; comparative fit index = .99; Tucker–Lewis index = .90). The dotted line represents the non‐significant associations. The bold line represents significant associations that test our hypothesis. *p < .01. **p < .001.

**Figure 2:**
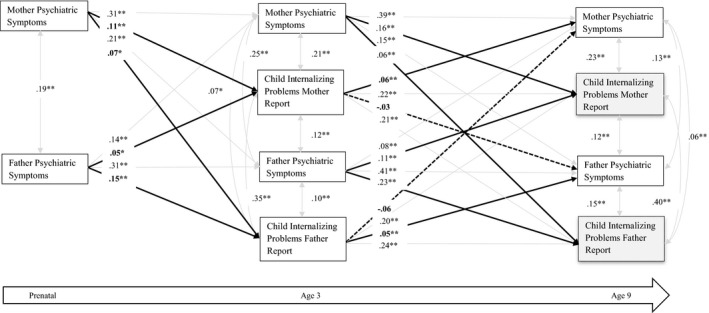
Structural equation modeling of parental psychopathology and child internalizing problems. Numeric values are standardized path regression coefficients. The models are adjusted for parental age, ethnicity, education, child sex and age, smoking, alcohol consumption and prenatal parental psychopathology reported by mother and father (root mean square error of approximation = .02; comparative fit index = .99; Tucker–Lewis index = .92). The dotted line represents the non‐significant associations. The bold line represents significant associations that test our hypothesis. *p < .01. **p < .001.

Separately for Externalizing (Figure 1) and Internalizing (Figure 2), standardized linear regression coefficients were used in the cross‐lagged panel SEM analyses at different ages of assessment and different informants. The model included paths from prenatal maternal and paternal BSI to child Externalizing and Internalizing scores at subsequent time points (e.g., ages 3 and 10), as well as from child Externalizing and Internalizing scores at earlier time points to maternal and paternal BSI scores at subsequent time points (e.g., ages 3 and 10). We also estimated the coefficients representing stability paths from one parental BSI score to the subsequent parental BSI scores and from one child Externalizing score to the next. Each SEM model also included cross‐sectional correlations between parental BSI and child Externalizing scores; and all paths and covariances were freely estimated. Furthermore, we evaluated in stratified analyses whether there are differences in the bidirectional associations between parent and offspring psychopathology determined by child gender.

To test whether the within‐rater parent‐to‐child psychopathology are statistically different from the cross‐rater associations, Wilson estimates of 84% confidence intervals of the estimates were compared. In contrast to 95% confidence intervals, 84% confidence lead to a probability of overlap of approximately 5% (Julious, 2004), and therefore, if confidence intervals of two estimates do not overlap, they differ significantly (Julious, 2004; Payton, Greenstone, & Schenker, 2003).

Including both maternal and paternal reports in the same SEM model addresses (dis)agreement between informants and thus was our model of choice. However, we also present an extended version of this classical SEM, namely an auto‐regressive latent trajectory model with structured residuals (ALT‐SR; Berry & Willoughby, 2017; Curran et al., 2014) to better disaggregate the within‐ and between‐person associations of parental and offspring psychopathology differences. This model addresses both stability and change of psychopathology over time. The ALT‐SR model incorporates latent growth curve modeling and correlates the latent intercepts (the estimated population mean level and residual between‐person variance) and slopes (the between‐person variance associated with the rate of the change) across parental BSI scores and child outcomes. We estimated the between‐person associations by the latent growth measures and (co) variances. The covariance between our latent growth factors extracts the disaggregated between‐person parameters (represented by Ѱstandardized below), thus pushing the remaining within‐person variance into the residual auto‐regressive and cross‐lagged portion of the model. The cross‐lagged and auto‐regressive components of this model represent within‐person deviations from one’s own typical trajectory.

In ALT‐SR models, we first tested the within‐person auto‐regressive associations among parental BSI scores and child outcomes as well as the between‐person intercepts and slopes. It is important to understand differences between the within‐ and between‐person associations and how estimates can be biased when the variance in not disaggregated (Berry & Willoughby, 2017; Hoffman, 2015; Hoffman & Stawski, 2009). We specified random intercepts and a linear slope with grow rates free to vary across individuals for both BSI scores and child outcomes (Internalizing and Externalizing problems). Next we tested reciprocal associations between parental BSI scores and child outcomes. All ALT‐SR models were constraint to be equal over time. Covariance between the two intercepts (parental BSI scores and child outcomes) and the two slopes (parental BSI scores and child outcomes) were estimated. In each model, we adjusted for all previously mentioned confounders at baseline, and for all models the intercepts and slopes were regressed on each of the confounders.

The SEM and ALT‐SR models are conducted to enable causal inference, but they cannot demonstrate causality in the way that a randomized controlled trial can do. Hence, we avoid causal language in the Results and Discussion sections and infer causality very cautiously, as suggested by Hernán (2018).

Since parental and offspring psychopathology were measured by both mothers and fathers and repeatedly over time, invariance was tested using ∆*χ* ^2^ *—*chi‐square difference tests (Satorra & Bentler, 1994) to determine whether bidirectional estimates of the associations were statistically different between mothers and fathers. Three separate sets of constraints were imposed. The models were first fit with the bidirectional associations between parental and offspring psychopathology estimated freely, that is not constrained. Then two sets of models were constrained to be equal for mothers and fathers. One set constrained the associations from parental BSI to child Externalizing scores to be equal over time, and the other set constrained the associations from child Externalizing to parental BSI scores be equal over time. For example, the stability between parental BSI scores prenatal and age 3 was constrained to be equal to the stability of parental BSI scores between ages 3 and 10. Comparison of the free versus constrained path models indicates whether the associations for mothers and fathers are different. As ∆*χ* ^2^ *—*is sensitive to sample size, we also examined the difference in comparative fit index (∆CFI) and root mean square error of approximation (∆RMSEA; Chen, 2007).

To address the missing data, we used full information maximum likelihood (FIML) to account for the missing data. FIML avoids uncertainties from estimating data and provides unbiased estimates of missing parameters in large sample size while retaining natural variability in missing data (Enders, 2010). Thus, each participant contributes to the data they have available at each time point to the likelihood function and no participants are removed from analyses through listwise deletion. In addition, we compared our findings with and without FIML procedures (i.e., listwise deletion was employed) and found no evidence that our estimates were biased by the missing data. The data were analyzed using SAS 9.4 for descriptive statistics and SEM, and Mplus 8 (Muthén & Muthen, 2017) for ALT‐SR.

Root mean square error of approximation ≤ .05, and the CFI and Tucker–Lewis index (TLI) ≥ .90 were taken to indicate good fit in the SEM models. When comparing the estimated SEM models, goodness‐of‐fit was also evaluated using chi‐square (Kline, 2015).

## Results

Parental and child characteristics are presented in Table 1. Mothers were on average 31 years at the birth of the child, fathers 33 years. In total, 28% of mothers and 25% of fathers had a non‐Western national origin. Tables S1 and S2 show the correlations, means, and standard deviations between parental BSI measures and child Externalizing and Internalizing scores, respectively. Longitudinal correlations for child Externalizing and Internalizing problems were consistently higher for the same informant ratings (e.g., mothers' ratings at different time points) versus cross‐informant ratings (e.g., mothers’ ratings at one time point and fathers’ ratings at another time point). Also, correlations between parental psychopathology and child Externalizing or Internalizing problems were consistently larger if scores were based on the same informant (e.g., mothers’ self‐reports of her psychopathology and mother‐reported child behavior problem) versus different informants (e.g., mothers’ self‐reports of her psychopathology and father‐reported child behavior problem).

**Table 1:** Baseline Characteristics of Study Sample (*N* = 5,536)

	Mother	Father
Age, *M* (*SD*)	30.9 (4.8)	33.3 (5.3)
Ethnicity		
Dutch (%)	62.6	67.9
Other Western (%)	9.3	6.9
Non Western (%)	28.1	25.2
Educational level		
High (%)	52.4	54.8
Middle (%)	28.9	25.7
Low (%)	18.7	19.5
Alcohol use during pregnancy		
No consumption during pregnancy (%)	37.4	
Until pregnancy recognized (%)	13.8	
Continued occasionally (%)	38.4	
Continued frequently (%)	10.4	
Smoking during pregnancy		
No smoking during pregnancy (%)	79.8	
Until pregnancy recognized (%)	12.5	
Continued during pregnancy (%)	7.6	
Gender (% boy)	49.5	
Age, years, *M* (*SD*)	10.1 (0.6)	

Note

Numbers denotes children included in one or more analyses. Values are frequencies for categorical and means and standard deviations (*M* ± *SD*) for continuous measures.

Figure 1 shows the SEM of the bidirectional associations between parental BSI and child Externalizing scores reported by mothers and fathers. Results indicated good fit to the data (RMSEA = .01, CFI = .99, and TLI = .90). The standardized coefficients obtained in the SEM analyses are presented in the figure, with straight lines representing significant associations and dotted lines non‐significant associations. The autoregressive coefficients showed that parental BSI scores were moderately stable and yet sufficiently variable over time to model change. Similar association patterns over time were observed between the repeatedly measured Externalizing and also the Internalizing scores (Figure 2). Both maternal and paternal BSI scores were associated with child Externalizing scores at ages 3 and 10, with children exposed to higher parental BSI scores having higher Externalizing scores. The association of maternal and paternal psychopathology with child outcome was consistently stronger if rated by the same rater than by the other parent, that is, cross‐informant. The reverse associations, those from child to parent are also shown in Figure 1. When reported within‐rater, the associations between child Externalizing scores on parental psychopathology were similar to those for parent‐to‐child associations. No paths testing the associations between child Externalizing scores during childhood were associated with parental psychopathology across‐rater.

Comparing the within‐ and across‐rater associations by calculating 84% confidence intervals showed that, for example, the association of mother‐reported child Externalizing scores at age 3 with mother‐reported BSI scores at age 10 (84% CI: [.04, .05]) differed from the association of mother‐reported child Externalizing scores with father‐reported BSI scores at age 10 (84% CI: [−.06, −.05]), as the CIs do not overlap. Similarly, the associations of mother‐reported child Internalizing scores at age 3 with mother‐reported BSI scores at age 10 (84% CI [.03, .04]) differed from the association of mother‐reported child Internalizing scores with father‐reported BSI scores at age 10 (84% CI [−.02, −.04]). Based on these comparisons (not shown), there is evidence for a difference between the effect estimates of the within‐rater parent‐to‐child psychopathology and the cross‐rater associations.

Next, we tested the bidirectional association of parental psychopathology and child Internalizing scores with a similar model. Again, RMSEA = .02, CFI = .99, and TLI = .92, showed a good fit to the data (see Figure 2). The estimates for Internalizing were very similar to the results for child Externalizing scores. Prenatal maternal and paternal BSI scores were consistently related to higher child Internalizing scores at ages 3 and 10 as reported by mothers and fathers. For child Internalizing scores, results remained consistent over time, with higher Internalizing scores associated with parental psychopathology over time, but only within‐rater. In summary, both mothers’ and fathers’ child Externalizing and Internalizing reports were associated with their level of psychopathology symptoms over time.

**Figure 3:**
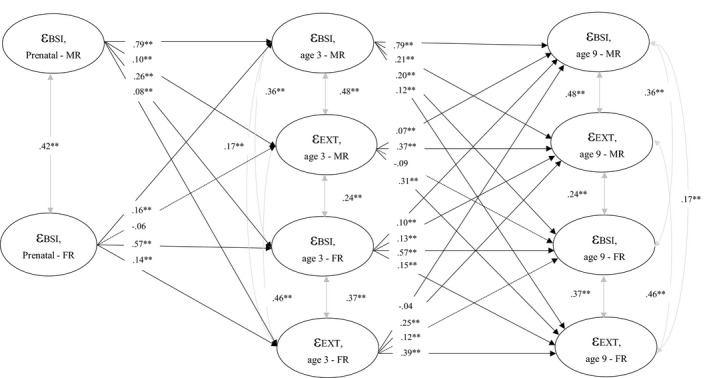
Autoregressive latent trajectory model with structured residuals, disaggregating the within‐ and between‐person associations of parental and offspring psychopathology. Numeric values are standardized path coefficients. The dotted lines represent the non‐significant associations. The control variables (parental age, ethnicity, education, child sex and age, smoking, alcohol consumption and prenatal parental psychopathology reported by mother and father) are not shown in the figure for the ease of interpretation. ε (epsilon) = residual variance; BSI = parental psychopathology; EXT = child externalizing problems; MR = mother report; FR = father report. Full parameter estimates can be found in Table S4. *p < .01. **p < .001.

Figure 3 shows the results of the ALT‐SR models for parental BSI and Externalizing scores reported by mothers and fathers. Intercept and slope factors represented by latent growth models indicated moderate to strong associations for between‐person maternal and paternal BSI and child Externalizing scores. Specifically, we observed that higher initial levels of maternal BSI scores were associated with higher initial levels of Externalizing scores (Ѱstandardized = 2.94, *p* < .001), as well as higher initial levels of paternal BSI scores were associated with higher initial levels of Externalizing scores (Ѱstandardized = 2.83, *p* < .001). In the final within‐person cross‐lagged model, we observed bidirectional associations between maternal and paternal BSI and child Externalizing scores, whereby higher levels of maternal and paternal BSI scores than typical (i.e., higher than the individual mean) were associated with higher levels of child Externalizing scores at the next time point, and vice versa. Our final model resulted in good fit to the data (CFI = .97, RMSEA = .003).

**Figure 4:**
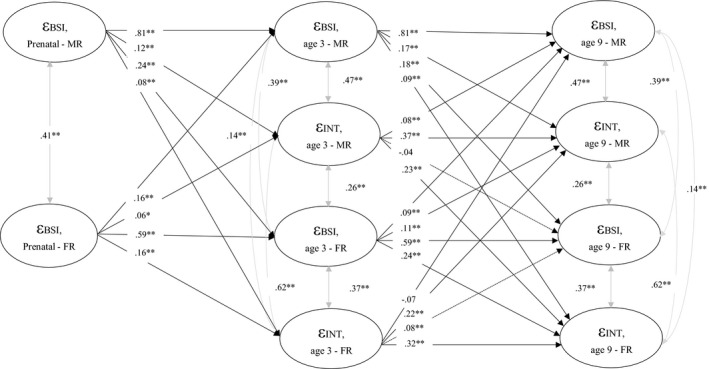
Autoregressive latent trajectory model with structured residuals, disaggregating the within‐ and between‐person associations of parental and offspring psychopathology. Numeric values are standardized path coefficients. The dotted lines represent the non‐significant associations. The control variables (parental age, ethnicity, education, child sex and age, smoking, alcohol consumption and prenatal parental psychopathology reported by mother and father) are not shown in the figure for the ease of interpretation. ε (epsilon) = residual variance; BSI = parental psychopathology; INT = child internalizing problems; MR = mother report; FR = father report. Full parameter estimates can be found in Table S4. *p < .01. **p < .001.

Next, the covariance between random intercepts for maternal BSI and child Internalizing scores (Ѱstandardized = 2.63, *p* < .001), and paternal BSI and child Internalizing scores (Ѱstandardized = 2.55, *p* < .001) were modeled. When evaluating the within‐person reciprocal effects, we found that higher levels of both maternal and paternal BSI scores were associated with higher levels of child Internalizing problems than their typical levels at the next time point, and vice versa. Our final model resulted in good fit to the data (CFI = .96, RMSEA = .009; Figure 4).

In addition, we tested whether bidirectional paths coefficients significantly differed across mothers and fathers using ∆*χ* ^2^ *—*chi‐square difference tests. Invariant bidirectional paths estimated freely for both mothers and fathers provided a good fit to the data. When bidirectional path models were constrained to be equal across mothers and fathers, neither the paths from parental BSI to Externalizing scores nor the paths from Externalizing to parental BSI scores differed between mothers and fathers. Next, the invariant bidirectional paths estimated freely for parental BSI and child Internalizing scores provided a good fit to the data. As before, when equality constraints were set, neither the patterns for BSI to child Internalizing scores nor the paths from child Internalizing to BSI scores, differed significantly between mothers and fathers (Table S3). However, father and mother reports independently predicted child behavioral problems; that is, each parent contributed unique information.

Stratified analyses showed that our findings regarding the bidirectional associations between parent and offspring psychopathology did not differ by gender of the child. Likewise, the parent‐to‐child psychopathology associations did not vary by gender of the child (results not shown).

## Discussion

This large population‐based study examined the bidirectional associations between parent and offspring psychopathology reported by mothers and fathers up to age 10. Specifically, we examined the associations between parent and offspring psychopathology by leveraging recent advances in modeling longitudinal associations that disaggregate within‐ and between‐person associations. Ratings provided by both mothers and fathers enabled us to also examine differences between gender of the parents in the bidirectional associations of parent and offspring psychopathology. We highlight three main findings. First, parental psychopathology and child externalizing or internalizing problems were consistently associated within‐raters but not across‐raters. The magnitude of parent‐to‐child associations were stronger than the reverse associations. Second, maternal and paternal reports of psychopathology did not differ between within‐ and between‐rater in the bidirectional associations with the child outcomes. Third, bidirectional associations between parent and offspring psychopathology were found at both the within‐ and between‐person levels. Overall, finding bidirectional associations only within‐rater of parent and offspring psychopathology but not across‐rater suggests that the observed within‐rater child‐to‐parent associations probably reflect shared‐rater variance.

### Bidirectional Associations

The present findings provide consistent evidence of a bidirectional association of parent and offspring psychopathology within‐raters, but not across‐raters. That is, the associations of both maternal and paternal reports with child psychopathology over time appear to have been significantly affected by shared‐rater variance. We did not find support for the notion that the bidirectional associations differed significantly between mothers and fathers. The use of different informants to test bidirectionality in one model enabled us to study the associations between parental psychopathology and child externalizing and internalizing problems within‐ and across‐raters, both of which are important for informing research and clinical practice.

### Within‐ and Between‐Person Findings

The current study extends the prior work indicating the observed bidirectional associations between parental and offspring psychopathology by showing that these associations were evident at both within‐ and between‐person levels. The between‐person level showed that both mothers and fathers who reported higher levels of psychopathology also reported higher levels of child Externalizing or Internalizing problems, and vice versa. The within‐person associations showed that, for a given person, changes in one’s typical level of psychopathology over time were associated to the subsequent child Externalizing or Internalizing problems, and vice versa. That is, in our study the observed associations are prominently expressed both as within‐ and as between‐person associations. This suggests that parental psychopathology and changes in child problem behavior occur due to individual differences in trajectory (i.e., between‐person associations), as well as due to changes arising at the within‐person level. Although the associations of both maternal and paternal reports of child externalizing or internalizing problems with parental psychopathology were observed within‐raters but not across‐raters over time, the model yielded significant between‐person associations among all variables (i.e., significant covariances between intercepts). This suggests that mothers or fathers who reported higher levels of psychopathology also reported higher levels of child problem behavior. Furthermore, although prior research has shown that parent psychopathology may undermine to children’s healthy development (Breaux et al., 2014; Connell & Goodman, 2002), these earlier studies used a between‐person approach that does not partition variance at multiple levels of analysis. Our model is an improvement on prior work as it takes into account within‐ and between‐person effects, and specifically examines how deviations from one’s typical level of psychopathology can affect changes in child problems at subsequent time points, and, further, how child problems can affect changes in psychopathology.

### Parent‐to‐Child Associations

Our results indicated that parental psychopathology was associated with offspring psychopathology. This was true for both externalizing and internalizing problems in the child. The parent‐to‐child associations were stronger for within‐rater associations than for cross‐rater associations, but both sets of associations were significant. That is, coefficients were smaller when self‐rated psychopathology of one parent was used to associate child psychopathology as rated by the other parent than when child psychopathology was rated by the same parent.

Theoretical models (e.g., Dodge, 1990) suggest four mechanisms could explain the observed associations between parental psychopathology and child outcome, including (a) genetic transmission; (b) pregnancy‐specific effects, which imply that, for example, maternal psychopathology may lead to direct physiological changes impacting fetal development; (c) exposure to parents’ maladaptive affect, behavior, cognitions, which can lead to dysfunctional modeling; and (d) contextual stressors, such as family disruption, that are related to the development of child problem behavior (Goodman & Gotlib, 1999). For example, disadvantaged parents may have less time to facilitate children’s social activities. Although we cannot conclude which of these mechanisms contributed most to these associations, results of our study help guide the mechanistic understanding. In particular, we argue that the mechanism “b” is less likely compatible with our results, as we did not observe meaningful difference between maternal‐ and paternal‐reported psychopathology in the bidirectional associations with child outcomes. Moreover, we carefully controlled for contextual stressors.

Furthermore, our results are only partly consistently with the theoretical model that has emphasized the transactional processes of change in the development of child problems (Sameroff, 1975). Sameroff’s (1975) theory emphasized the development of the child as a product of the continuous dynamic interactions of the child and the experience provided by his or her social settings. Children and their parents mutually affect one another when children elicit particular types of responses from their parents and when parents’ behavior induces children to behave in particular ways in the future. We confirmed the parent‐to‐child associations, but child‐to‐parent associations were only observed within‐rater. Moreover, the within‐person change accounted for a substantial part of the variance observed. Because early childhood is a time of tremendous learning and growth, younger children may be more susceptible to parental influence than older children (Maccoby, 2000). Conversely, as children develop, their capacity to impact characteristics of their environments increases. For example, given that parents (and their behaviors) represent a central component of this environment, children’s ability to engage in meaningful interactions with parents will be greater than of infants, or might be bidirectional (Perlman & Ross, 1997). It is also possible that both parent and child effects will weaken over time because children become less depended on parents over time and more influenced by peers and other social factors (Cicchetti, 2016; Ingoldsby & Shaw, 2002), but they are still influenced by child‐ and parent‐driven processes that combine to shape the home environment.

### Child‐to‐Parent Associations

Findings for child psychopathology in association with subsequent parent psychopathology were generally weaker than those in the parent‐to‐child direction. That is, when parents rated their child’s problems and then later rated their own psychopathology, the associations were significant but generally smaller than those for the parent‐to‐child associations across the same time periods. These child‐to‐parent associations were not affected by type of problem (externalizing vs. internalizing). However, these associations were significantly affected by shared‐rater variance. This can be seen in the fact that cross‐rater child‐to‐parent associations were generally very weak and not significant. Consequently, parents, who are often involved in their children’s presenting problems, are not necessarily neutral reporters. Specifically, parental psychopathology may narrow the parent’s tolerance for child problem behavior, such that minor behavioral problems are misperceived as major issues. The narrowed band of tolerance found among parents with psychopathology symptoms would result in a lowered threshold for child problems, which in essence is based on a distorted perception of child problem behavior (Fergusson, Lynskey, & Horwood, 1993). Alternatively, parents’ negative perception of their children not supported by other informants must not directly follow from parental misperceptions of child problem behavior. Rather, high parental rates of reported child problems could stem from problematic interactions in family, rather than from parental negative perceptions.

### Shared‐Variance Issues

That the longitudinal associations between child and parent psychopathology were largely observed only within and not cross‐rater, could in principle reflect three factors, namely cross‐rater disagreement (Achenbach, 2006; De Los Reyes & Kazdin, 2005), information bias, and importantly, shared‐rater variance, which is a particular form of information bias (Collishaw, Goodman, Ford, Rabe‐Hesketh, & Pickles, 2009; Geiser, Eid, Nussbeck, Courvoisier, & Cole, 2010; Jaffee & Poulton, 2006). First, the differences in the associations from child‐to‐parent psychopathology could depend on the rater, but associations across raters were absent or weak independent of the parental perspective on child behaviors (Achenbach, 2006; Achenbach, McConaughy, & Howell, 1987; De Los Reyes & Kazdin, 2005). It is likely that mothers and fathers have different kinds of relationships with children that evoke different behaviors (Achenbach, 2006). For example, fathering practices in terms of coaching and team sport typically differ from maternal parenting, which more occurs at home (Gavanas, 2004). However, we found no differences between the associations of child psychopathology with mothers’ and fathers’ problem rating. Consequently, the pattern of the within‐ and across‐rater associations was similar in mothers and fathers. Second, the informants’ reports about his/her own psychopathology or the psychopathology their child can be distorted. For example, parent’s reports on their child’s problems could be biased by their own emotional problems, or by poor understanding of the questions. If this distortion is related (indirectly, for example due to unmeasured background factors) to the outcome assessment of the child, this could introduce a spurious relation and constitute information bias. Third, these discrepancies in the bidirectional associations between one rater and across raters can reflect shared method variance bias. This type of information bias occurs if an external factor influences both the ratings obtained for the parent and the child. It is very likely that social desirability, negative affectivity, and acquiescence (tendency to agree) affect ratings to some degree (Spector, 2006). These factors suggest that if a parent rates both his/her own psychopathology and child problem behavior, inflated shared variance is likely to occur. Characteristics of the instrument, for example the related paper and pencil setting of the CBCL and BSI measures or similar item wording, can also allow bias (Podsakoff, MacKenzie, & Podsakoff, 2012). Thus, the shared method variance can result from the construct (e.g., the psychopathological trait), the method and importantly the informant. However, in most studies, the likelihood of shared‐rater variance is considerable since they used the same informant both on measures of parental psychopathology and on measures of child problem behaviors. The findings of the current study extend the literature by showing associations and possible bias by shared method variance for both parents and different types of psychopathology.

### Gender Findings

We found no evidence for parental gender differences between the bidirectional associations of parent and offspring psychopathology. Previous studies showed that mothers more frequently serve as primary caretaker in the family (Pleck & Hofferth, 2008), and spend more time with children relative to fathers (Lamb, 2010; Pleck, 2012). However, we observed no differences in the associations of maternal and paternal psychopathology with child problems. This may indicate that the effect of psychopathology is independent of the time spent with the child or that genetic factors largely determine intergenerational transmission of psychopathology.

Similarly, investigating child gender, we found that the inclusion of a bidirectional association for parent and offspring psychopathology did not differ by gender of the child. This is in line with results of the two meta‐analyses that focused specifically on interparental agreement in ratings of their child’s problems indicating that gender of the child did not moderate discrepancies in mother and father ratings of their child’s problems (Achenbach et al., 1987; Duhig, Renk, Epstein, & Phares, 2000). However, discrepant reports with some evidence of moderation by child gender have also been published. For example, Choe et al. (2014) found that boys with suboptimal self‐regulation exposed to high levels of maternal depressive symptoms showed a higher risk of school‐age behavior problems.

### Externalizing Versus Internalizing Problems

A final important finding of our study is that we found similar patterns of associations for the SEM models for externalizing versus internalizing problems, suggesting that both mothers and fathers respond to their children’s externalizing and internalizing problems similarly. Externalizing and internalizing problems represent two different types of children’s problems, but have considerable shared variance due to a general psychopathology factor contributing to both (Caspi et al., 2014). This may be why we did not detect differences regarding how parental psychopathology could differentially be associated by their child’s externalizing and internalizing problems.

### Strengths and Limitations

Several limitations of our study should be noted. First, only parents reported on their own problems and child problem behavior. We do not know what the results would be if other informants on problem behavior were obtained, such as father ratings on maternal psychopathology, mother ratings on paternal psychopathology, clinician’s ratings on parental psychopathology and teacher‐, clinician‐ or (if the child were old enough) self‐reports on child problem behavior. Second, it is also likely parental psychopathology could reflect biological vulnerability to offspring problem behaviors. Biological vulnerability can be based on shared genetically characteristics (Goodman et al., 2011), which may increase offspring susceptibility to develop emotional and behavioral problems. The strengths of the study lie in its large population‐based sample and the SEM approach to longitudinal measurement testing of a bidirectional association among two parents’ and child psychopathology. Furthermore, we included both maternal and paternal reports on child problem behavior and therefore could examine both separate maternal to child and paternal to child models, as well as combined parent‐to‐child associations. This indicates that psychopathology in fathers and mothers are equally associated with offspring psychopathology. Our analyses also suggest that the contributions of the parents are independent of each other, thus not due to spousal resemblance. This underlines the importance of involving fathers in research (Parent, Forehand, Pomerantz, Peisch, & Seehuus, 2017). Another strength of this study underlies the use of ALT‐SR model to simultaneously consider between‐person associations among parental and offspring psychopathology (e.g., mean levels growth rates), with simultaneously modeling bidirectional associations between these variables as they manifest within‐person over time.

Our findings have important implications for future research and clinical practice. First, they suggest that targeting parental psychopathology among high‐risk parents may be effective in reducing both child externalizing and internalizing problems during childhood. As the associations of child‐to‐parent psychopathology are small, interventions aimed at parental psychopathology that include a child component would likely be only marginally more effective. Yet, in the long term, such interventions could certainly enhance the parental and other family member’s well‐being even if not measurable in terms of parental psychopathology. Overall, our findings show that psychopathology of parents is a crucial target of prevention and intervention efforts for children with developmental problems. However, whether interventions for children with psychopathology should largely focus on parents with psychiatric problems, only on children, or on both depends on the child age, the developmental status, and cognitive capacities. Moreover, any intervention to interrupt the negative transactional processes between parental psychopathology and child emotional and behavioral problems would need to be aware of other social influence and complexities determining when and in whom to intervene.

To conclude, our findings suggest that bidirectional associations of parent and offspring psychopathology can be consistently detected only within‐rater but not across‐rater. Moreover, the within‐person levels of psychopathology explained substantial variance of child problems, and vice versa. Child Externalizing and Internalizing problems were both predicted by earlier parental psychopathology. In contrast, child psychopathology is only weakly associated with later parental psychopathology, and with cross‐rater associations generally not significant. Child gender do not affect these associations. The findings highlight the importance of shared‐rater variance, suggesting that using the same rater inflates the associations between parental psychopathology and child outcomes in both directions.

## Supporting information


**Table S1.** Correlation Coefficients, Means and Standard Deviations Between Parental Psychopathology and Child Externalizing Behavior

**Table S2.** Correlation Coefficients, Means and Standard Deviations Between Parental Psychopathology and Child Internalizing Behavior

**Table S3.** Differential Effects of Bidirectional Associations Between Parental and Offspring Psychopathology: A Comparison Between Mothers and Fathers (*N* = 5,536)

**Table S4.** Autoregressive Latent Trajectory Model With Structured Residuals: Bidirectional Associations Between Parent and Offspring Psychopathology (*N* = 5,536)
