# Supplementary material for: From Parent to Child to Parent: Associations Between Parent and Offspring Psychopathology
Source: Child Dev. 2020 Aug 26;92(1):291–307. doi: 10.1111/cdev.13402 (PMC7891374; doi:10.1111/cdev.13402)
Supplement: Supplementary file 1 — Table S1. Correlation Coefficients, Means and Standard Deviations Between Parental Psychopathology and Child Externalizing Behavior [file CDEV-92-291-s001.docx]

Supplementary Material

Supplementary Table 1. Correlation Coefficients, Means and Standard Deviations between Parental Psychopathology and Child

Externalizing Behavior

|  |  |  | 1 | 2 | 3 | 4 | 5 | 6 | 7 | 8 | 9 | 10 | 11 |
| --- | --- | --- | --- | --- | --- | --- | --- | --- | --- | --- | --- | --- | --- |
|  | M | (SD) |  |  |  |  |  |  |  |  |  |  |  |
| Child externalizing problems |  |  |  |  |  |  |  |  |  |  |  |  |  |
| 1 Age 1.5, mother report | 10.6 | 6.73 | **-** |  |  |  |  |  |  |  |  |  |  |
| 2 Age 3, mother report | 8.13 | 6.14 | .56** | **-** |  |  |  |  |  |  |  |  |  |
| 3 Age 3, father report | 9.20 | 6.45 | .36** | .56** | **-** |  |  |  |  |  |  |  |  |
| 4 Age 9, mother report | 3.59 | 4.56 | .32** | .44** | .32** | **-** |  |  |  |  |  |  |  |
| 5 Age 9, father report | 3.71 | 4.61 | .23** | .32** | .40** | .61** | **-** |  |  |  |  |  |  |
| Parental psychopathology |  |  |  |  |  |  |  |  |  |  |  |  |  |
| 6 Prenatal, mother report | .24 | .32 | .20** | .19** | .11** | .14** | .07** | **-** |  |  |  |  |  |
| 7 Prenatal, father report | .13 | .19 | .11** | .10** | .11** | .07** | .13** | .24** | **-** |  |  |  |  |
| 8 Age 3, mother report | .15 | .25 | .22** | .31** | .19** | .20** | .11** | .35** | .14** | **-** |  |  |  |
| 9 Age 3, father report | .13 | .23 | .18** | .21** | .29** | .16** | .20** | .19** | .26** | .39** | **-** |  |  |
| 10 Age 9, mother report | .22 | .29 | .17** | .21** | .10** | .25** | .13** | .33** | .11** | .45** | .21** | **-** |  |
| 11 Age 9, father report | .16 | .24 | .06** | .11** | .18** | .17** | .25** | .09** | .23** | .16** | .37** | .25** | **-** |

**Correlation is significant at the 0.01 level (2-tailed).
