# Supplementary material for: From Parent to Child to Parent: Associations Between Parent and Offspring Psychopathology
Source: Child Dev. 2020 Aug 26;92(1):291–307. doi: 10.1111/cdev.13402 (PMC7891374; doi:10.1111/cdev.13402)
Supplement: Supplementary file 3 — Table S3. Differential Effects of Bidirectional Associations Between Parental and Offspring Psychopathology: A Comparison Between Mothers and Fathers (N = 5,536) [file CDEV-92-291-s003.docx]

Supplementary Table 3. Differential Effects of Bidirectional Associations between Parental and Offspring Psychopathology: A Comparison

between Mothers and Fathers (N=5,536).

|  | Compared with | χ^2^ | *df* | RMSEA | CFI | *p* | ∆χ^2^ | ∆*df* | ∆RMSEA | ∆CFI | *p* |
| --- | --- | --- | --- | --- | --- | --- | --- | --- | --- | --- | --- |
| **Externalizing problems, mother and father reports** |  |  |  |  |  |  |  |  |  |  |  |
| Model 1: Parent reported psychopathology and child  externalizing problems |  | 269.8 | 89 | .009 | .97 | .65 |  |  |  |  |  |
| Model 2: Parental psychopathology 🡪 child externalizing problems | Model 1 |  |  |  |  |  | 128.9 | 44.5 | .003 | .02 | .42 |
| Model 3: Child externalizing problems 🡪 parental psychopathology | Model 1 |  |  |  |  |  | 122.4 | 44.5 | .006 | .01 | .51 |
|  |  |  |  |  |  |  |  |  |  |  |  |
| **Internalizing problems, mother and father reports** |  |  |  |  |  |  |  |  |  |  |  |
| Model 1: Parent reported psychopathology and child  internalizing problems |  | 284.7 | 89 | .006 | .99 | .38 |  |  |  |  |  |
| Model 2: Parental psychopathology 🡪 child internalizing problems | Model 1 |  |  |  |  |  | 132.1 | 44.5 | .001 | .03 | .24 |
| Model 3: Child internalizing problems 🡪 parental psychopathology | Model 1 |  |  |  |  |  | 118.9 | 44.5 | .004 | .02 | .37 |

Note: Model 1: In the first model all bidirectional associations of parent and child (internalizing or externalizing) problems were estimated freely. Model 2: In the second model all

associations from parental BSI scores to child (externalizing or internalizing) problems were constrained equal. Model 3: In the third model all associations from child (externalizing or

internalizing problems) problems to parental BSI scores were constrained equal. The models are adjusted for parental age, ethnicity, education, child sex and age, smoking, alcohol

consumption and prenatal parental psychopathology reported by mother and father. The following indexes are reported: χ^2^ = chi-square difference test; RMSEA = Root mean square error

of approximation; CFI = Comparative fit index; Δ = change in statistical values.
