# Supplementary material for: From Parent to Child to Parent: Associations Between Parent and Offspring Psychopathology
Source: Child Dev. 2020 Aug 26;92(1):291–307. doi: 10.1111/cdev.13402 (PMC7891374; doi:10.1111/cdev.13402)
Supplement: Supplementary file 4 — Table S4. Autoregressive Latent Trajectory Model With Structured Residuals: Bidirectional Associations Between Parent and Offspring Psychopathology (N = 5,536) [file CDEV-92-291-s004.docx]

Supplementary Table 4. Autoregressive latent trajectory model with structured residuals: Bidirectional Associations

between Parent and Offspring Psychopathology (N=5,536).

|  | Externalizing problems^a^ |  | Internalizing problems^b^ |
| --- | --- | --- | --- |
| ALT-SR effects | Parameter estimate (SE) |  | Parameter estimate (SE) |
|  |  |  |  |
| Within-person cross-laggs |  |  |  |
| BSI_t+1_ on CBCL_t_ - MR | 0.40 (0.09)** |  | 0.43 (0.08)** |
| BSI_t+1_ on CBCL_t_ - FR | 0.36 (0.07)** |  | 0.33 (0.07)** |
| Auto-regressive |  |  |  |
| BSI_t+1_ on BSI_t_ - MR | 0.79 (0.10)** |  | 0.81 (0.11)** |
| CBCL_t+1_ on CBCL_t_ - MR | 0.37 (0.07)** |  | 0.37 (0.06)** |
| BSI_t+1_ on BSI_t_ - FR | 0.57 (0.09)** |  | 0.59 (0.08)** |
| CBCL_t+1_ on CBCL_t_ - FR | 0.39 (0.06)** |  | 0.32 (0.06)** |
| (Co)variances (between- person) |  |  |  |
| BSI_int_ with CBCL_int_ - MR | 2.94 (0.15)** |  | 2.63 (0.14)** |
| BSI_int_  with CBCL_slope_ - MR | 1.95 (0.13)** |  | 1.72 (0.11)** |
| CBCL_int_  with BSI_slope_ - MR | 2.25 (0.15)** |  | 2.27 (0.13)** |
| BSI_slope_ with CBCL_slope_ - MR | 1.13 (0.09)** |  | 1.19 (0.09)** |
| BSI_int_  with CBCL_int_ - FR | 2.83 (0.14)** |  | 2.55 (0.14)** |
| BSI_int_  with CBCL_slope_ - FR | 1.87 (0.11)** |  | 1.54 (0.12)** |
| CBCL_int_  with BSI_slope_ - FR | 1.99 (0.12)** |  | 2.15 (0.14)** |
| BSI_slope_ with CBCL_slope_ - FR | 1.10 (0.10)** |  | 1.13 (0.11)** |
| Residual (co)variances |  |  |  |
| BSI _ε_*_it1 –_* _ε_*_it3_* - MR | 4.21 (0.45)** |  | 4.07 (0.44)** |
| CBCL_ε_*_it1 –_* _ε_*_it2_* - MR | 3.89 (0.37)** |  | 3.83 (0.38)** |
| BSI _ε_*_it1 –_* _ε_*_it3_* - FR | 4.15 (0.43)** |  | 3.87 (0.39)** |
| CBCL_ε_*_it1 –_* _ε_*_it2_* - FR | 3.26 (0.33)** |  | 3.12 (0.32)** |
| Fit statistics |  |  |  |
| *χ*^2^ | 438.53 |  | 429.77 |
| *df* | 144 |  | 144 |
| RMSEA | .003 |  | .009 |
| SRMR | .009 |  | .010 |
| CFI | .97 |  | .96 |

Note: ALT-SR = autoregressive latent trajectory with structured residuals. All models presented here

are final models with all within correlation auto-regressive paths and cross-lagged associations being

estimated. Variables on the left side of an ‘on’ statement are the depended variable at t + 1. Those on

the left side represent the independed variables. BSI = Parental psychopathology; CBCL = child

Internalizing or Externalizing problems; In the table subscripts identify time of measurement.

For example, a single ‘*t’* indicates paths were constraint to be equal over time; ‘t +1’ represents an

outcome for a specific unidirectional path at the next time point. Subscript ‘int’ indicates latent intercept

(mean level) to obtain between-person parameter estimates. Subscripts with an epsilon (ε*it*1) indicate

residual variance from Time 1 to Time n.

^a^ includes estimates of mother- or father-reports psychopathology and child externalizing problems.

^b^ includes estimates of mother- or father-reports psychopathology and child internalizing problems.

RMSEA = root mean square error of approximation; SRMR = standardized root mean square

error of approximation; CFI = comparative fit index. MR = mother report; FR = father report.

*p<0.01. **p<0.001.
